# Towards the identification of transmission pathways and early detection of *Enterococcus cecorum* infection in broiler chickens

**DOI:** 10.1016/j.psj.2024.104224

**Published:** 2024-08-16

**Authors:** K. Watson, L. Arais, S. Green, P. O'Kane, M. Kirchner, T. Demmers, C. Commins, R. Smith, G. Cordoni, I. Kyriazakis, A. Schock, M.F. Anjum

**Affiliations:** ⁎ School of Agricultural Sciences and Practice, Royal Agricultural University, Cirencester, GL7 6JS, United Kingdom; † Animal and Plant Health Agency, Addlestone, KT15 3NB, United Kingdom; ‡ Applied Group, Chesterfield, S40 1DU, United Kingdom; § Slate Hall Veterinary Services, Metheringham, Lincoln, LN4 3HX, United Kingdom; # Department of Pathobiology and Population Sciences, Royal Veterinary College, Hatfield, AL9 7TA, United Kingdom; ǁ Institute for Global Food Security, Queen's University Belfast, Belfast, BT9 5DL, United Kingdom; ¶ Animal and Plant Health Agency, Lasswade, EH26 0PZ, United Kingdom

**Keywords:** broiler, Enterococcus cecorum, detection, transmission, behavior

## Abstract

*Enterococcus cecorum* (**EC**) infection is an emerging endemic disease in UK and global broiler poultry with major economic impact and welfare concerns. There are significant research gaps with regards to EC pathogenesis, source of infection, transmission routes and early detection of disease, which this study aimed to address. In this prospective study, 725 environmental samples were collected from 4 broiler farms (A–D) the day before chick placement (d 1) and through the subsequent crop (d 7, 14, and 21). Cecal swabs were collected from birds that died of natural causes during the study period. A sample of birds that had been found dead or were culled for health reasons, were presented for post-mortem and samples were taken from lesions for EC culture. DNA was extracted from all environmental samples and EC detected using a qPCR and MALDI-TOF. Two EC isolates from diseased birds were inoculated on concrete slabs and incubated at 23°C and 32°C followed by swabbing of concrete culturing and determination of EC cfu at defined time points. Alongside environmental and bird sampling commercially available, smart camera systems were installed in selected houses on each farm to monitor bird activity and distribution. No EC outbreak occurred during the study, however, it was detected by qPCR in 215/725 (29.7 %) of all samples collected. Also, EC DNA was detected on average in 37% of samples collected on d 1, with approx. 88% of samples from chick paper being positive. Despite this, it was only cultured from 3 ceca samples and joint fluids of two infected birds from farm B on d 14 and 21. The survival experiments using isolates from infected chickens showed EC can survive on concrete for at least 21 d. This study provides invaluable insights into transmission pathways and tenacity of EC. Further studies are needed to determine strain characteristics in relation to their ability to cause disease and to further elucidate the sources of infection on poultry farms.

## INTRODUCTION

*Enterococcus cecorum* (EC), originally named *Streptococcus cecorum* (Devriese et al., 1983), was considered an enteric commensal bacterium of adult poultry. Since its first isolation from lame commercial broiler chickens (Devriese et al., 2002, Wood et al., 2002), EC has been recognized as an important pathogen in many countries with a substantial impact on bird welfare and the economy (De Herd et al., 2009; Stalker et al., 2010; Dunnam et al., 2023; Souillard et al., 2022). Farm mortality rates in affected flocks can be up to 15%, with additional losses due to high rejection rates at slaughterhouse (Borst et al., 2012; Jung and Rautenschlein, 2014; Borst et al., 2017). The main clinical signs are lameness, hock sitting and hindlimb paralysis; and post mortem lesions include pericarditis, splenomegaly, septic arthritis, spondylitis and osteomyelitis in the 6th thoracic vertebrae or femoral head (Wood et al., 2002; Borst et al., 2017, Dunnam et al., 2023).

Substantial gaps in knowledge remain with regards to the source of infection, routes of transmission and EC strain pathogenicity and virulence. Since EC was first isolated from lesions from British broilers (Wood et al., 2002), no systematic investigation has been published on the epidemiology of EC in the UK.

The pathogenesis of EC disease remains to be fully elucidated. Disease-associated isolates have been shown to be genetically distinct from intestinal commensal strains (Huang et al., 2023). Oral transmission has been described as the most likely route of infection (Martin and Martin, 2011) and pathogenic EC will colonize the chicken gut prior to bacteremia and systemic infection (Borst et al., 2017). The development of clinical disease including osteomyelitis may be multifactorial, with risk factors including immunosuppression due to a variety of stress factors, alterations of the intestinal microbiome, and factors influencing bone homeostasis (Wideman, 2016).

Transmission of pathogenic strains through environmental sources has been suggested but not proven. The recent development of a quantitative PCR (**qPCR**; Jung et al., 2017) enabled detection of EC in the farm environment (Grund et al., 2022; Tessin et al., 2023), although attempts to culture EC from environmental samples were unsuccessful (Robbins et al. 2012; Borst et al., 2017, Grund et al., 2022).

To protect bird welfare and avoid major economic losses, there is a need to detect pathogenic EC infection earlier in the production cycle. Initially, clinical signs and increased mortality were usually detected from 28 d onwards in the broiler sector (De Herdt et al., 2009; Stalker et al., 2010) and early detection was rarely reported (Borst et al., 2017). The infection is commonly detected from around d 10 in the production cycle and usually presents with septic arthritis or pericarditis (Borst, 2020).

In order to address the research gaps identified, we carried out a prospective observational study on 4 broiler farms, from placement to depletion. The aim was to characterize any potential EC outbreak and to collect and analyze environmental and cecal samples to investigate EC transmission routes and environmental reservoirs of infection. Furthermore, we aimed to investigate EC survival abilities on concrete surfaces by conducting in-vitro survival experiments. This work was complimented by using an established method for behavioral monitoring capable of detecting changes in flock activity, which may be associated with lameness.

## MATERIALS AND METHODS

### Farm Selection and Management Systems

Four commercial UK broiler farms (Farms A–D) with either 2 (Farms A and D) or 4 (Farms B and C) poultry houses were chosen for the study. Farms were selected with a focus on ensuring a diverse range of performance expectations including their historical performance. Farms C and D both had at least 1 clinically significant outbreak of EC disease that required antibiotic treatment within the previous year. All farms housed Ross 308 broilers, representing a variety of UK chick suppliers, feed mills and processors, all were compliant with the Red Tractor farm assurance scheme and national law. Houses were stocked with 40,000 to 50,000 birds depending on the house size (Farm A: 3,160 m^2^. Farm B: 2,254 m^2^. Farm C: 2,507 m^2^. Farm D: 2,507 m^2^), growing to a maximum stocking density of 38 kg/m^2^. All farms operated a mixed sex, all-in all-out production system, with newly hatched chicks being placed into disinfected houses followed by a 37 to 42 d growth cycle (crop). Partial depopulation (thinning) occurred after wk 4. All birds were housed in climate-controlled houses and kept on fresh wood shavings or rape straw pellets.

Birds had ad libitum access to commercial broiler feeds containing coccidiostat and monitored via a weigher-tipper. Water was continuously supplied via a storage tank connected to the main water supply. Feed and water intakes were recorded on a Fancom panel (Fancom BV, Penningen, The Netherlands) and the data was accessed by Optifarm (Chesterfield, UK) through its remote monitoring AI platform for advice and support. All flocks received vaccines for Infectious Bronchitis Virus and Infectious Bursal Disease Virus. Environmental data was collected across all sites, including measurements of temperature (Fancom temperature sensor SF-7, Fancom, Panningen, The Netherlands), humidity (RH sensor Fancom Set L/N T05726, Fancom, Panningen, The Netherlands), CO_2_ levels (Fancom 4270025 CO_2_ Sensor, Fancom, Panningen, The Netherlands) and ventilation. Daily mortality and culling rates were recorded, and bird weights were recorded via 2 automatic scales per house (Fancom Lumina 47, Fancom, Panningen, The Netherlands) in addition to a weekly manual weigh of a representative sample of the flock (Table 1).

**Table 1:** Cumulative mortality (%), culls due to leg abnormalities (%) and bird body weights (**BW**, g) of trial farms A-D throughout the trial crop with data shown for d 7, 14, 21, 28, and 35.

		Cumulative mortality (%)					Cumulative leg cull (%)	BW (g)				
		Day										
Farm	House	7	14	21	28	35	35	7	14	21	28	35
A	1	1.25	1.75	2.46	3.33	4.77	0.90	192	508	996	1658	2216
A	2	1.07	1.62	2.31	3.00	3.55	0.72	198	511	1068	1642	2278
B	1	1.56	2.37	3.29	4.21	5.06	0.46	189	507	1004	1551	2220
B	2	2.19	3.22	4.19	5.19	6.35	0.63	182	543	1001	1688	2320
B	3	1.43	2.37	3.57	4.72	5.7	0.65	188	487	965	1525	2250
B	4	1.82	2.8	4.19	5.54	6.33	0.71	187	503	1010	1583	2280
C	1	1.79	2.26	2.64	2.87	3.25	0.22	152	455	933	1550	2136
C	2	2.0	2.86	3.41	3.7	4.28	0.57	182	503	1009	1650	2240
C	3	1.44	2.09	2.55	2.80	3.36	0.48	182	512	1024	1620	2220
C	4	2.59	3.17	3.69	3.98	4.77	0.72	156	470	966	1550	2156
D	1	1.44	1.88	2.2	2.46	2.89	0.79	180	512	1033	1579	2135
D	2	1.13	1.46	1.75	2.06	2.41	0.30	169	499	989	1550	2190

Trials were approved by the Animal Welfare and Ethical Review Body (**AWERB**) of the Royal Agricultural University, although no live animals were used for research.

### On-Farm Sample Collection

Environmental samples were collected from a range of sources the day before chick placement (d 1) and on d 7, 14 and 21. All samples were collected using sterile sampling procedure using either swabs (ESwab, COPAN diagnostics, Murrieta, California) or universal sampling containers (Sterilin, Thermo Scientific , Loughborough, United Kingdom). All samples for DNA extraction were frozen on arrival at -20 °C (litter and chick paper) and -80°C (Swab samples) until processing. Samples for culturing where plated on arrival, except for farms C and D, where samples had to be frozen at -80°C for operational reasons. For this, 20% glycerol was added to the e-swab Amies medium, following COPAN diagnostics recommendations. After farm disinfection and the day before chick placement (d 1), floor swabs (n = 7) were taken at each sampling event following a W-shaped trajectory across the house, to allow coverage of the entire house (Supplementary Figure 1). Swabs were also taken from the house walls (n = 6) and randomly selected chick boxes (n = 6) on chick arrival. In addition, papers lining these chick boxes (chick papers) were collected (n = 6). Swabs were taken from the surface of drinker cups and from inside of the downstream ends of drinker lines at d -1, 7, 14, and 21. Pooled litter samples were collected at d 7, 14, 21 following the same W-shaped trajectory. In addition, 4 pooled samples of unused litter from each farm (n = 16) were tested for EC presence acting as a negative control. A variable number of cecal swabs were collected from birds that were found dead by farm staff.

### Veterinary Inspection and Post Mortem Analysis

All farms were under the care of poultry-specific veterinary practices, providing standard care and investigations in response to health or performance issues. This included veterinary site visits or post-mortem submissions to the veterinarian's offices as deemed necessary. Where performed, macroscopic intestinal examination was conducted, including assessment of tone and contents as described by Teirlynck et al. (2011). In addition to standard veterinary care, a sample of birds (n = 280, 137 male and 143 female birds) that had been found dead (n = 51), or were culled for any health reasons (n = 229), were presented to Slate Hall Veterinary Services (Lincoln, UK) for examination at approximately 2, 3, and 4 wk of age. Each sample was selected from a single day's mortality and culls. No healthy birds were submitted for this study and decomposed birds (as assessed by carcass color and condition) were excluded from the examination. A routine, systematic post-mortem examination was performed, with emphasis on the locomotor system, including hindlimb bones and joints, and free thoracic vertebrae. Weights, sex and any observed lesions, as well as microbiological data, were systematically recorded in a bespoke practice database (Insight, Slate Hall Veterinary Services, Lincoln, UK). A total of 84 bacteriological samples (up to 5 per house) were aseptically collected from lesions that suggested bacterial infection. These included joint fluid; pericardium; bone marrow; spleen and heart. Swabs were plated on Blood and MacConkey agar (Thermo Fisher Scientific PB0115A and PO0148A, Loughborough, UK) and incubated for up to 48 h at 37°C in microaerobic conditions using a candle jar. *Enterococcus* spp. were identified by colony morphology, catalase activity and Gram stain characteristics, and further identification by API Strep (API 20 Strep, Biomerieux, Basingstoke, UK) and/or Matrix-assisted laser desorption/ionization (MALDI-TOF, Bruker Ltd, Coventry, UK).

### Culturing of EC From Ceca and Environmental Samples From Farm Trial Samples

Buffered peptone water (**BPW**) was used to prepare 10-fold serial dilution of swabs and environmental samples to achieve 50 to 150 colonies/plate. Dilutions were plated on Columbia blood agar supplemented with horse blood, 10 mg/l of colistin sulphate and 5mg/L of oxolonic acid (Streptococcus Selective Supplement, Oxoid, Basingstoke, UK) and incubated overnight at 37°C in a 5% CO_2_ enriched atmosphere (Grund et al., 2022) using CO_2_ gas generator sachets (Oxoid, Basingstoke, UK). Three to 5 colonies with morphology of EC (small greyish colonies with alpha-hemolysis) were selected and identified using MALDI-TOF (Randall et al., 2015).

### Detection of EC Using Quantitative PCR

***DNA extractions***. DNA was extracted from swab samples using the DNeasy Blood and Tissue Kit (Qiagen, Manchester, United Kingdom). Swabs were centrifuged for 1 min at 4,000 rpm and the liquid Amies medium was pelleted by centrifuging for 1 min at 12,000 rpm. The pellet was suspended in 250 μL of the medium which was then used to extract DNA according to the manufacturer's instructions.

A subset of 0.5 grams of pooled litter samples and chick paper were transferred into a microbead tube. Briefly, pooled litter samples were mixed thoroughly to ensure a homogenous distribution of the material and chick paper was processed by randomly selecting and cutting six fragments with fecal contamination from each paper and pooling them into the microbead tube. DNA was extracted from litter and each chick paper, using the NucleoSpin DNA Stool kit (Mackerey-Nagel, Düren, Germany) with no assay modifications. Samples were extracted in batches of 11 with a negative control (250 μl sterile H_2_O) in each batch. Samples were randomized before processing, to avoid the risk of processing bias.

***Assay sensitivity and specificity***. A qPCR assay targeting a 60 base pair sequence of the EC 16S rRNA gene was used. Details on sensitivity and specificity have been described previously for DNA from cultured isolates (Jung et al., 2017). The qPCR was tested on a panel of six *Enterococcus* species: *E. faecalis, E. faecium, E. gallinarum, E. hirae, E. casseliflavus and E. durans*, in addition to *E. cecorum*, which were included as positive controls, to determine specificity for detecting *E. cecorum* (Supplementary table 1).

As DNA was extracted directly from environmental swabs and sources, a series of spiking experiments were conducted to provide further information on detection limits. Swabs and sterilized qPCR negative wood shavings (litter) were inoculated with 50 μL of undiluted and 10-fold serially diluted and quantified EC culture. Dilutions ranged from 10^−9^ to 10^9^ resulting in approximately 1.08 to 1.08 × 10^9^ copies per swab/litter samples. Dilutions were also plated in duplicate on CNA agar and incubated for 24 h to enable counting of cfu's.

***Assay set up***. DNA was extracted from samples as described above and tested with qPCR (AriaMx Real-Time PCR System, Agilent technologies, Stockport, United Kingdom). All qPCR plates were processed using the same primers, TaqMan probe and cycling conditions previously described (Jung et al., 2017). Each 20 μL qPCR reaction contained 2μL of sample, 1μL of each primer (Forward primer: 5′-ACAGGTGCTAATACCGCATAAT-3′; reverse primer: 5′-CCCACCAACTAGCTAATGCAC-3′) and 0.5μL of TaqMan probe (5′-FAM-ACCGCATGGTAGATGGATGAAAGGC-BHQ1–3) (each at a concentration of 10 pmol/μL), 5.5 μL of nuclease free water and 10 μL of PerfeCTa qPCR ToughMix (Quanta Biosciences, Inc., Beverly, MA). All samples were processed in triplicate and every plate had standard curve samples and a non-template control (nuclease free water). As a positive control, EC strain IS12-14,619 (Supplementary table 1) was used. Samples were classified as positive for EC when all 3 qPCR replicates amplified with an average Ct-value of ≤ 35, with Ct-values ranging from 10 to 35.

### Automated Behavioral Monitoring of Flock Activity and Distribution

Commercially available intelligent cameras systems were installed on all trial farms to monitor and record flock activity patterns and distribution, for example, cluster movements and spread. Farm A were fitted with the greengage ALIS system (Greengage international, Greengage Agritech Ltd, Edinburgh, United Kingdom) offering a welfare score based on assumed norms in activity and spread using thermal camera technology, with two cameras fitted in each house. Farms B, C and D were fitted with the eYeNamic poultry behavior monitor (Fancom BV, Panningen, The Netherlands). Houses 2, 3 and 4 of farm B had 6 EyeNamic V2 cameras fitted in each house, whilst farms C and D had 2 EyeNamic V3 cameras in two of the houses. Both systems use bespoke algorithms to detect the change in color of pixels between subsequent images of each camera. The number of pixels changed, combined with the location, is used as a measure for activity and distribution. Both systems saved data at least every 5 min using their dedicated software (ALIS and Farmmanager, respectively).

### Viability of EC on Concrete Surfaces at 23°C and 32°C

In a controlled laboratory experiment, EC strains EC2/CEC22 (isolated from cecal contents through APHA surveillance) and D22-2347-1 (isolated from farm B joint fluid) (Supplementary Table 1) were used to investigate survival on concrete surfaces. One cm thick concrete slabs were disinfected and autoclaved. Slab surfaces were divided into 4 cm^2^ squares and inoculated with 100 μL of enumerated EC cultures (5.2 × 10^7^ and 2.3 × 10^7^ cfu's for EC2/CEC22 and D22-2347-1, respectively) in duplicate and incubated at 23°C and 32°C for the duration of the trial (21 d). The inoculated area was swabbed immediately after inoculation, at 8 h and 24 h, followed by swabbing on d 2, 3, 5, 7, 9, 14, and 21. Swabs were streaked in duplicate on CNA agar and incubated as described above. Cfu data was collected and EC was identified using the qPCR.

### Statistical Analysis

All statistical analysis was conducted in R-studio 2023.09.01 (R Core Team, 2023). Distribution of the data was tested using the Shapiro-Wilkes test. Subsequently Kruskall-Wallis H test was used to investigate statistical differences between EC loads by sample type overall and for each farm. Multiple pair wise comparisons were then conducted using the Dunn test and *p*-values where adjusted using the Benjamini-Hochberg method to reduce the risk of type 1 error. Friedman's test was used to investigate changes of bacterial load from Farms A and B, over time. The low number of positive samples from farms C and D throughout the crop did not allow for meaningful statistical analysis. In order to calculate survival probabilities and statistical differences of survival between EC strains at each temperature, survival analysis was used. The “survival” and “survminer” packages were used to plot corresponding Kaplan-Meier survival curves for each temperature.

## RESULTS

### Farm Performance, Health Status, and Post Mortem Results

Observed mortality rates, including culling for lameness and weights for all farms are presented in Table 1. None of the 4 farms included in this study had a clinically significant EC outbreak during the study period. By 20 d of age, birds on farm A showed an increase in daily mortality and culling rates due to lameness. Following a diagnosis of femoral head necrosis and colisepticemia by the site's primary veterinarian, with nonhemolytic *Escherichia coli* cultured from visceral tissue cultures, a 5-d course of amoxicillin was prescribed for both houses. On farm B, mortality rates were higher than the other farms (Table 1) and poor feed conversion ratio was recorded (4 points higher than the average of the 5 previous production cycles for this site). Poor weight uniformity, with wet litter and poorly formed droppings where observed in all houses at d 14/15. Investigation by the farm's primary veterinarian revealed changes consistent with nonspecific poor intestinal health (such as poor tone and abnormal intestinal contents) on post-mortem examination. This was initially managed by supplementation including essential oil-based alternative products. After further deterioration of intestinal health was noted by the farm's primary veterinarian in houses 3 and 4 on d 21, a course amoxicillin was prescribed. From the trial-specific postmortem examinations and microbiological testing of tissue swabs, EC was cultured from joint fluid from one bird in house 1 and another from house 2 at 21 d from Farm B. Both joints had excessive quantities of abnormal fluid indicative of septic arthritis. Although mortality rates were generally higher from Farm B than on the other sites (Table 1), the total culling rate for lameness did not exceed 0.71% in any house. Daily culling rates were on average between 0.01% and 0.02% and did not exceed 0.052% on any single day.

On farm D, drops in growth rate, bird activity and feed and water consumption were observed after 35 d following thinning, findings from the farm's primary veterinarian were consistent with non-specific poor intestinal health for example, poor tone, mucus, abnormal contents.

Other findings noted across the remaining birds examined in the trial-specific post mortem examinations included conditions consistent with *E. coli, Staphylococcus* spp., *Enterococcus faecalis* and *Enterococcus hirae* infection; poor intestinal health, gizzard erosions coccidiosis, and cardiovascular disease such as ascites and sudden death syndrome.

### Detection and Quantification of EC in Cecal and Environmental Samples by Culture and qPCR

Three EC isolates were obtained from cecal swabs, collected from birds which died naturally or had been culled on welfare grounds in houses 3 (n = 2) and 4 (n = 1) on farm B. No EC was cultured from environmental samples.

**Figure 1:**
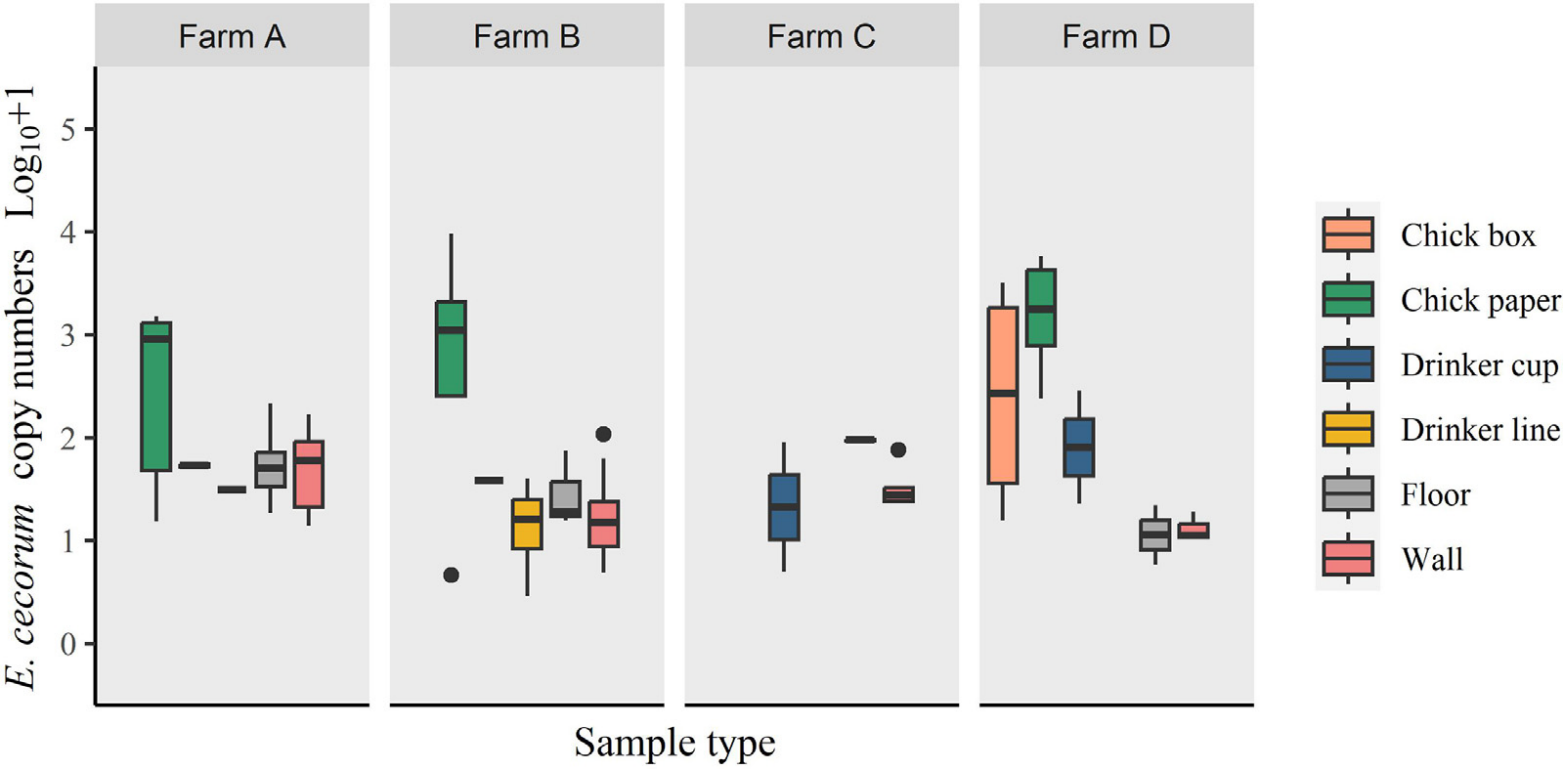
Log_10_ + 1 *Enterococcus cecorum* DNA copy numbers of positive samples determined by qPCR and detected on floor, wall and chick box swabs and chick paper samples, on the day prior to chick placement (d 1) on the 4 monitored farms A–D (n = 93).

**Figure 2:**
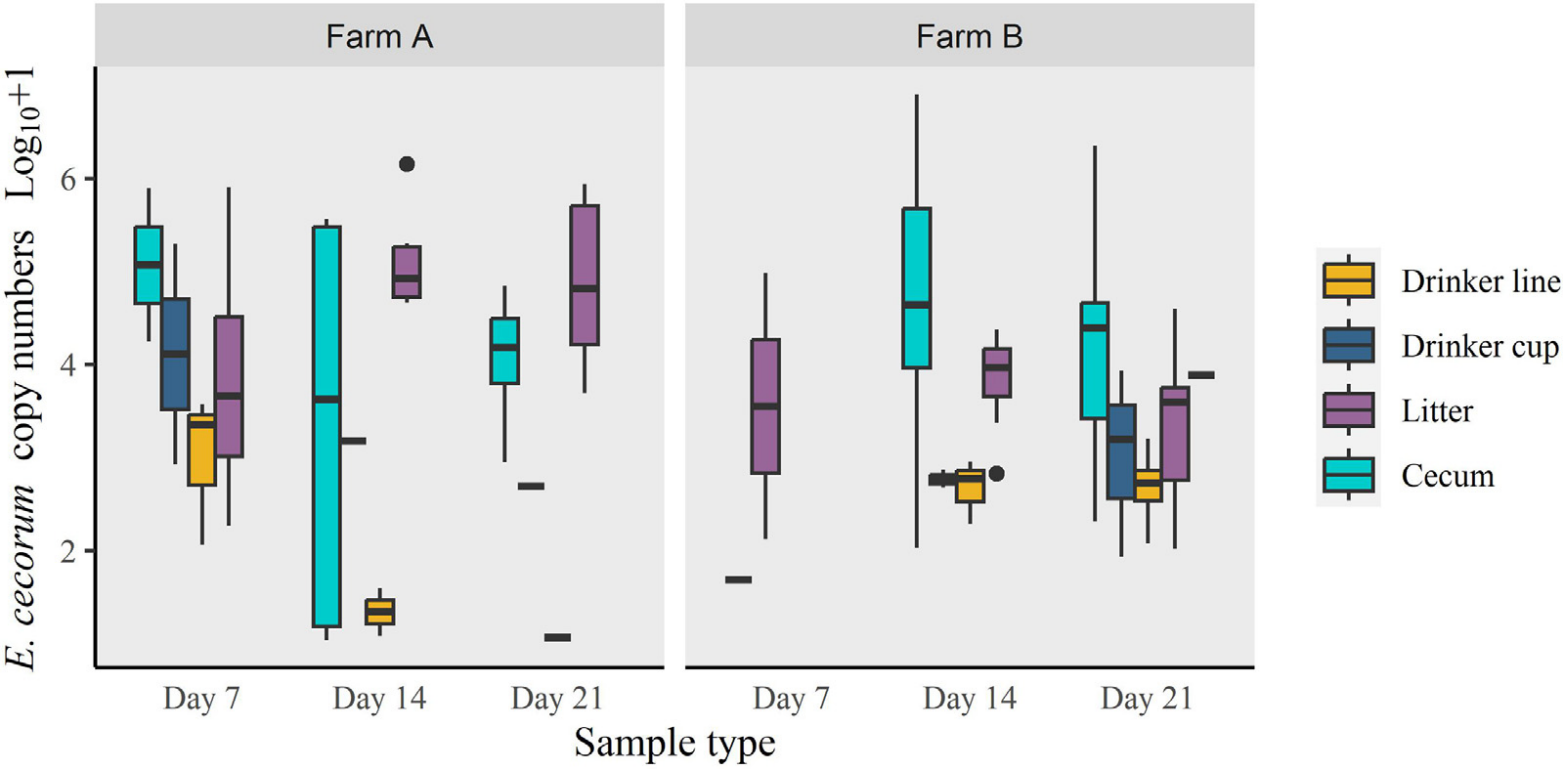
Log_10_ + 1 *Enterococcus cecorum* DNA copy numbers of positive samples determined by qPCR, in cecal samples, litter and swabs taken from drinker cups and drinker lines during d 7, 14 and 21 of the production cycle, on farms A and B (n = 110).

Validation of the qPCR showed that none of the six other *Enterococcus* species that were used to test the specificity of the qPCR (Supplementary Table 1), amplified. Experiments using samples spiked with EC determined the detection limit of the qPCR to be 1.07 × 10^1^ and 3.87 × 10^1^ copies of EC from swabs and litter samples, respectively. EC DNA was detected in 215/725 (29.7 %) samples. EC DNA was detected on all farms and sample types except in the 12 litter samples collected before chick placement (Table 2, Table 3). Of the 252 samples collected on the day prior to chick placement (d 1), 93 (23.9 %) amplified EC. On d 1 EC was detected on all chick papers from farms A, B and D, with EC copy numbers being significantly higher in this sample type compared to EC detected on drinker line (*P* = 0.009), or swabs from walls (*P* = 0.014) and floor (*P* < 0.001) (Figure 1). When comparing EC loads on sample types for each of the farms, no significant statistical differences were observed, except for farm D, where EC copies were significantly higher on chick paper than on wall swabs (*P* = 0.029). The remaining 473 samples analyzed originated from samples taken on d 7, 14, and 21 of the crop (Table 3). Notably, 99.0% and 88.3% of positive samples from farms D (n = 23) and C (n = 10), respectively, originated from d 1, with only 3 positive samples in total between d 7 and 21. On farms A and B, EC was detected throughout the crop (Table 3). On farm A, EC loads on cecal swabs and litter where significantly higher than loads on drinker line swabs (*P* = 0.040 and *P* = 0.045 respectively) but not drinker cups and litter. On farm B, EC loads where significantly higher on cecal swabs than on drinker lines (*P* = 0.002) and drinker cups (*P* = 0.036). EC loads on litter where significantly higher than EC loads from drinker lines (Figure 2). There was also variation of EC loads between sample types for each sampling day, and over time, however, there were no statistically significant difference, likely due to the low number of samples in each category.

**Table 2:** Detection frequency of *Enterococcus cecorum* DNA, assessed by qPCR from d 1, that is, the day prior to chick placement, from broiler house environmental samples and from chick paper and chick boxes at placement (**n = 252**), on all 4 farms (A–D)monitored.

	Chick paper	Chick box swabs	Floor swabs	Wall swabs	Drinker line swabs	Drinker cup swabs	Positive samples (% positives)
Farm-house	EC qPCR positive samples/tested samples						
Farm A	12/12	0/12	13/14	9/12	1/4	2/2	37/56 (66.1 %)
Farm B	4/4*	0/24	3/6	11/23	4/8	1/4	23/69 (33.3 %)
Farm C	0/4*	0/24	2/8	6/24	0/7	2/4	10/71 (14.1 %)
Farm D	12/12	4/12	2/14	3/12	0/4	2/2	23/56 (41.1 %)
Positive samples (% positive)	28/32 (87.5 %)	4/72 (5.56 %)	20/42 (47.6%)	29/71 (40.9 %)	5/23 (21.8 %)	7/12 (58.3 %)	93/252 (36.9 %)

Values represent numbers of positive samples against the total number of samples tested with percentage of positive samples in parenthesis.

⁎ Pooled sample from 4 houses. D 1 is the day prior to chick placement

**Table 3:** Detection frequency of *Enterococcus cecorum* DNA assessed by qPCR in litter and cecal samples (sampled on d 7, 14, 21) and swabs collected from drinker lines and cups (sampled on d -1, 7, 14, 21) on all 4 monitored farms (A–D).

Sample type										
	Drinker line					Drinker cup				
	Day					Day				
Farm	-1	7	14	21		-1	7	14	21	Positive samples (% positive)
A	1/4	3/4	2/4	1/4	7/16 (43.8 %)	2/2	2/2	1/2	1/2	6/8 (75. 0%)
B	4/8	1/7	3/7	4/6	12/28 (42.9 %)	1/4	0/4	3/4	3/3	7/15 (46.7 %)
C	0/7	0/8	0/8	0/8	0/31 (0.0 %)	2/4	1/4	0/4	0/4	3/16 (18.8 %)
D	0/4	0/4	0/4	0/4	0/16 (0.0 %)	2/2	0/2	0/2	0/2	2/8 (25.0 %)
Positive samples (% positive)	5/23 (21.8 %)	4/23 (17.4 %)	5/23 (21.7 %)	5/22 (22.7 %)	19/91 (20.9 %)	7/12 (58.3%)	3/12 (25.0 %)	4/12 (33.3 %)	4/11 (36.6 %)	18/47 (38.3 %)
Sample type										

	**Litter**					**Cecum**				
Farm	**Day**					**Day**				

	-1	7	14	21		-1	7	14	21	Positive samples (% positive)
A	NA*	12/14	7/14	9/14	28/42 (66.7 %)	NA	2/6	5/6	5/6	12/18 (66.7 %)
B	NA	2/27	7/28	19/28	28/63 (44.4 %)	NA	NA	5/9	8/11	13/20 (65.0 %)
C	NA	0/28	0/28	0/28	0/64 (0.0 %)	NA	1/12	0/12	0/12	1/36 (2.8 %)
D	NA	0/14	1/14	0/14	1/42 (2.4 %)	NA	0/6	0/6	0/6	0/18 (0.0 %)
Positive samples (% positive)	NA	14/83 (16.9 %)	15/84 (17.9 %)	28/84 (33.3 %)	57/211 (27.0 %)	NA	3/24 (12.5 %)	10/33 (30.3 %)	13/35 (37.1 %)	26/92 (28.3 %)

⁎ NA= No samples available. Values represent numbers of positive samples against the total number of samples tested, with percentage of positive samples in parenthesis. D 1 is the day prior to chick placement

### Viability of EC on Concrete Surfaces at 23°C and 32°C

**Figure 3:**
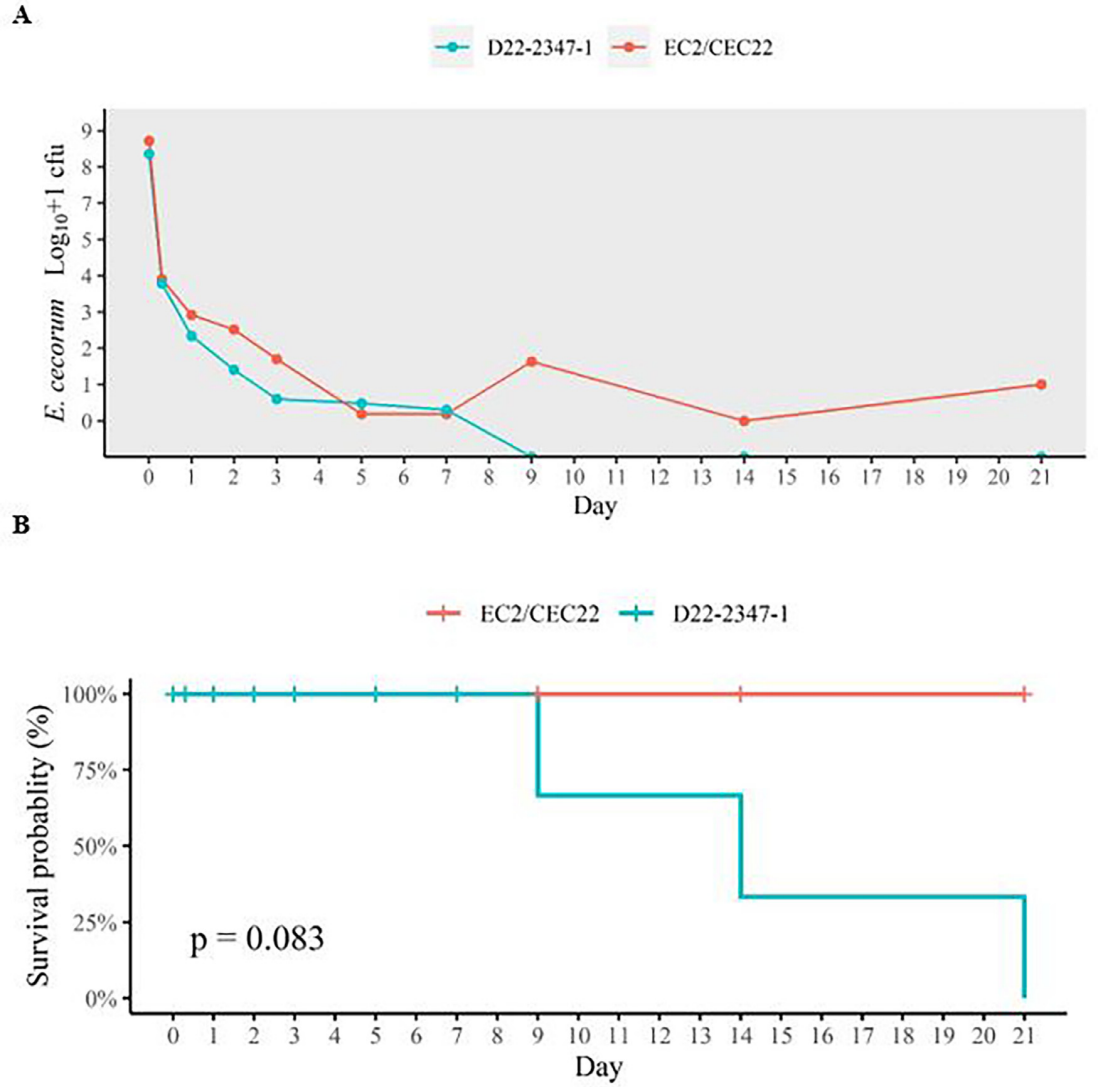
Log_10_+1 cfu (A) and survival probability (B) of *Enterococcus cecorum* recovered from concrete at 23°C from strains D22-2347-1 (Joint fluid, farm B) and EC2/CEC22 (Cecal isolate) at each sampling point (8 and 24 h, d 2, 3, 5, 7, 9, 14, 21). Time point zero represents *E. cecorum* log_10_+1 cfu's in 100 μL that were used to inoculate the concrete slabs.

**Figure 4:**
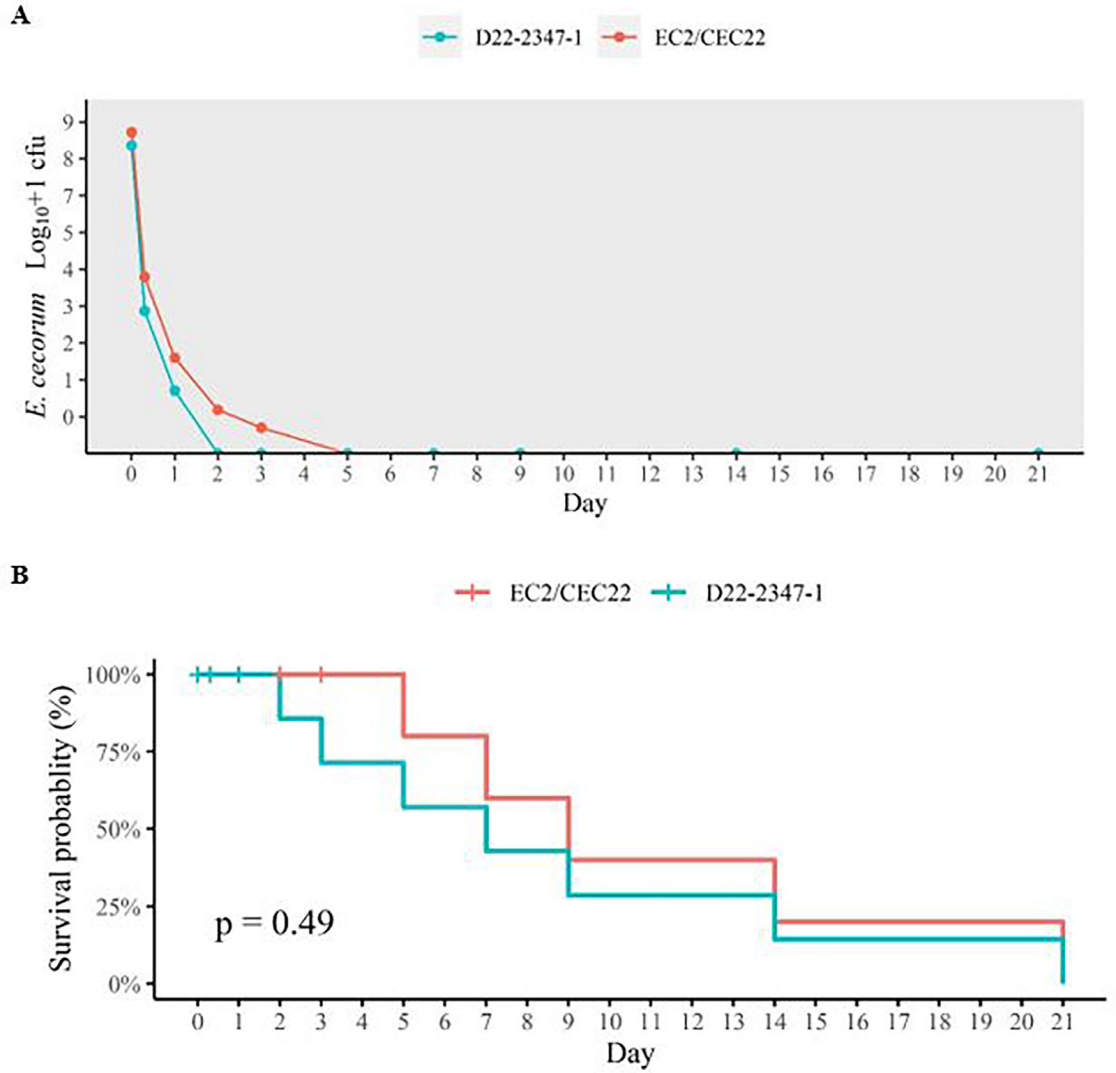
Log_10_+1 cfu (A) and survival probability (B) of *Enterococcus cecorum* recovered from concrete at 32°C from strains D22-2347-1 (Joint fluid, farm B) and EC2/CEC22 (Cecal isolate) on at each sampling point (8 and 24 h, on d 2, 3, 5, 7, 9, 14, 21).). Time point zero represents *E. cecorum* log_10_+1 cfu's in 100 μL that were used to inoculate the concrete slabs.

EC strains EC2/CEC22 isolated through the APHA scanning surveillance program from the cecal content of a EC diseased poultry, and D22-2347-1 isolated from farm B joint fluid, also from a diseased poultry, were used to test the tenacity of EC on concrete surfaces over 21 d at 23°C and 32°C. The number of cfu's from both strains declined sharply in the first 3 d of the trial at both temperatures (Figures 3A and 4A). Both isolates survived longer at 23°C than at 32°C, but there were no significant statistical differences in survival probability at each temperature for either strain (23°C: *p* = 0.083, 32°C: *p* = 0.49). However, at both temperatures cecal isolate EC2/CEC22-02 survived longer than isolate D22-2347-1 from joint fluid (Figure 3, Figure 4). At 23°C, isolate D22-2347-1 was recovered from concrete after 7 d, but not thereafter (survival probability 66.7%). Cecal isolate EC2/CEC22-02 was grown on the final day of the experiment (survival probability 100%) (Figure 3), indicating a survival time of at least 21 d.

### Automated Behavioral Monitoring of Flock Activity and Distribution

Due to technical issues with data transfer to the server, it resulted in very limited data being available for analysis for farm A, whilst an unidentified problem caused the data transfer between the EyeNamic cameras and FarmManager data collection to halt for a period of 2 wk (2–3) at farm C.

**Figure 5:**
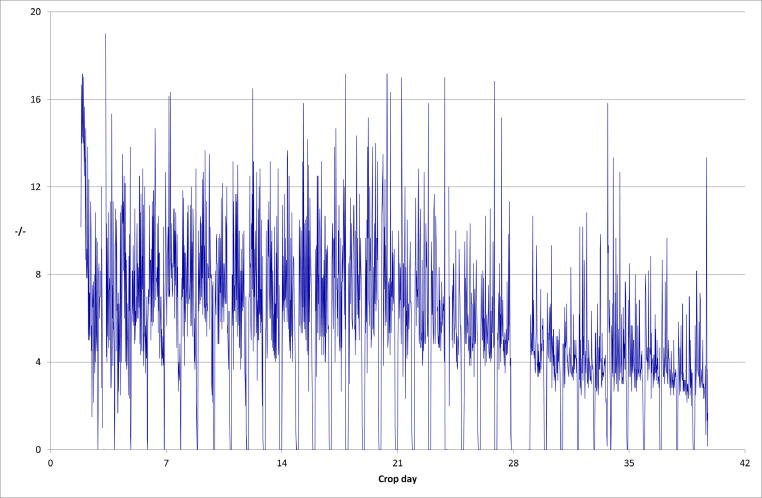
Example of flock activity showing patterns consistent with a healthy flock (Farm D/House 2). The activity was recorded by overhead cameras connected to the eYeNamic poultry behavior monitor (Fancom BV, Panningen, The Netherlands). The activity showed an initial spike after placement of the chicks and then a slow increase in activity value, followed by a decrease after approximately d 21. The distribution value increased to nominally 90% indicating an almost full coverage of the floor. Thinning, for example, removal of around 30% of the birds temporarily increased activity (for 3 d) and reduced the value for distribution. The upward spikes in activity are due to the twice daily walking of houses for inspection of birds by the farm manager. The downward spikes in activity are due to the dark periods.

No EC outbreak occurred on the farms used, therefore any changes in bird activity and distribution could not be reliably linked to EC infection and consequently EC detection and transmission could not be linked to the bird activity data. The automated behavior monitoring system showed mostly normal activity and distribution patterns, including where data was available for farms D (Figure 5 and Supplementary Figures 8–11) and C.

On farm B, houses 2, 3, and 4 followed activity and distribution patterns initially consistent with a healthy population (Supplementary Figures 2–7). Analysis of the activity data from farm B (Houses 3 & 4) revealed an unexpected small decrease in activity after d 15 (Supplementary Figures 5–7), suggesting a potential health concern. Simultaneously, there was a decrease in the distribution, suggesting a less than optimal spread of the birds, indicative of some clustering. This coincided with a noticeable drop in feed consumption from d 12. Activity remained subdued and a drop in water consumption was recorded on d 19. These findings coincided with investigation of and treatments for health issues unrelated to EC on d 15 and 21.

## DISCUSSION

This study aimed to investigate EC transmission routes, potential environmental reservoirs and early disease detection by collecting environmental samples and cecal swabs throughout the crop on 4 farms while monitoring bird activity and distribution using camera technology.

The main finding of this study is the detection of EC DNA in birds and the environment throughout the broiler production cycle on farms which, despite having a history of EC, did not develop into a significant outbreak attributable to EC. We also show, that EC DNA can be detected on broiler house walls and floors after farm disinfection. For the first time EC was detected on chick papers on 3 farms, providing evidence of possible vertical transmission from breeders and subsequent horizontal transfer via chick feces. Although EC is a dominant bacterium in the microflora of birds over 12 wk of age (Devriese et al., 1991), some EC have been isolated from the intestine of birds ranging from 1 to 6 weeks of age associated with subsequent disease (Gong et al., 2002; Borst et al., 2017). In contrast, EC strains from healthy birds has only been detected from 3 wk of age (Borst et al., 2017, Jung et al., 2018). Therefore, it is possible that EC can be vertically transmitted from the breeder flock, or acquired in the hatchery environment, but requires further investigation. Although *E. faecalis* and *E. faecium* infection of young poultry due to faecal egg contamination is well described (Rudy, 1991; Landman et al., 1999), vertical transmission of EC has yet to be definitively proven (Kense and Landman, 2011; Makrai et al. 2011, Robbins et al. 2012).

The occurrence of repeated subsequent infections in the same broiler house has previously led to suggestions of possible environmental reservoirs for EC (de Herdt et al., 2009). Grund et al. (2022) used qPCR to analyze swab samples collected from the start and end of drinker lines, cups and nipples in broiler houses during 2 consecutive crops. They detected EC in 62.5% of samples tested and on all surface types with the highest loads detected in drinker cups. Similarly, to our study, they also detected EC DNA in drinker lines after disinfection. Recently, Tessin et al. (2024) detected EC using qPCR in a range of environmental sources including drinker lines and drinker nipples, although no EC was found post disinfection. The results of this study add to existing evidence that EC in broiler drinking system could be a source of infection as all farms had a drinker line decontamination process prior to chick placement. This is supported by findings that *Enterococcus spp*. are able to survive in aquatic environments (Del Mar Lleo et al., 2005) for as long as 5 mo at 4°C and at room temperature. Biofilm formation has been described for other poultry-associated *Enterococcus spp*. (Woźniak-Biel et al. 2019), and this could potentially allow EC to survive and offer protection from sanitizers.

It remains unclear whether EC detected by qPCR in the farm environment was viable, due to unsuccessful culturing. Previous studies (Robbins et al. 2012; Borst et al., 2017, Grund et al., 2022) that attempted to detect EC in environmental samples using a culturing approach were unsuccessful.

Culturing protocols may lack sufficient sensitivity for EC isolation (Jung et al., 2018). In addition, the freezing of some culture samples may have affected EC isolation, although manufacturer recommended protocols were used. In addition, in our study, a low number of EC was detected by qPCR in most environmental samples and this was coupled with observations of a large numbers of other enterococcal species which readily grew on the current selective media. Therefore, it is possible that our culturing success of environmental samples was limited by both low EC numbers and competition from other organisms. Recently Tessin et al. (2023) reported the use of a chromogenic selective medium, for isolation of EC from a pooled fecal sample and carcass buckets on d 37 of the production cycle; this media could potentially improve isolation of EC in future when present at low levels.

EC was cultured successfully from cecal samples on farm B, where EC infection was confirmed in a single bird from houses 1 and 2, respectively. It is interesting to speculate that the carriage of EC above detection levels and very sporadic EC disease were because of the compromised intestinal health which affected these 2 houses. Mortality rates on farm B were also higher than on other farms, but below the UK Department for Environment, Food and Rural Affairs intervention trigger threshold of 7.37% (DEFRA, 2024), whereas the lameness culling rates (the usual sign of clinical EC infection) from farm B were on average under 0.019%, and did not exceed 0.038% on any day, hence were within normal range. In addition, 35 d weights from all farms were over 93% of the breed performance standard (Aviagen, 2022). This indicates a low prevalence of disease in Farm B without the development of a significant outbreak. As EC disease is likely to be multifactorial (Wideman 2016; Schreier et al., 2022), the overall higher mortality noted on farm B suggests other contributory predisposing factors may have been present. Combined with presence of intestinal EC, this may have contributed to the (albeit low prevalence) EC disease in houses 1 and 2.

The mortality rates from Farms A, C and D were within normal ranges for broilers, as reported from industry data (Avara Foods Animal Welfare report 2022-23; KFC 2021; Tabler et al. 2004). All of the other non-EC health conditions noted from post mortem examinations are common findings in dead birds from broiler flocks (Myers and Sander, 2019) and detailed investigation is out of scope for this paper.

It is interesting that although cecal EC DNA loads were high on both farms A and B, no EC was cultured on farm A and no EC associated disease was noted from the post mortem examinations of this study, or by the primary veterinarian. Although we cannot exclude that EC could have been present at a low prevalence in farm A, other competing pathogens present in farm A may also have “out-competed” EC during culture. However, mainly *E. coli* and *Staphylococcus* spp. were cultured from clinical samples, and *Enterococcus faecalis* was cultured from one joint (House 2, d 22). As only 3 to 5 colonies were picked per culture plate per sample for MALDI-ToF identification, EC could have been missed, if present in low numbers, requiring larger numbers to be tested in future. The amoxicillin treatment for *E. coli* infection could also have affected any potential EC and lowered the prevalence on farm A.

Tessin et al. (2024) reported detection of EC DNA post disinfection from two clinically healthy flocks in 6.7% of tested samples, in contrast to our study where 23.9 % of samples amplified. Our study showed very low concentration (estimated from Ct values in PCRs) of EC DNA in most sample types on d 1 on all farms, with bacterial DNA loads higher on chick paper and chick box swabs on farms A, B, and D. The results from farms A and D indicate that EC presence before chick placement, does not necessarily lead to disease, which is supported from results reported by Tessin et al. (2024). It is also possible that some DNA detected was from non-viable bacteria. Additionally, even though a higher EC load may be required to increase chances of infection, the infective dose required may depend on the virulence profile of EC strains, which currently remains unknown.

The sample sizes of birds examined at postmortem limited the ability to detect EC associated disease at low prevalence; this study was designed to detect clinically significant outbreaks of disease. Future refinements would include larger sample sizes and regular veterinary visits to farm to observe flocks for lameness and select these birds for examination. This was not possible due to biosecurity precautions as there were frequent outbreaks of H5N1 Highly Pathogenic Avian Influenza at the time of study (Ross et al., 2024).

It is possible that increased detection of EC on farms A and B were also related to hygiene status of these farms. It could possibly be attributed to differences in virulence characteristics of circulating strains, however this cannot be proven as no EC was cultured from Farm A. Also, the pathogenic mechanism of EC remains unknown.

Our survival experiment showed that a clinical EC strain, present in APHA archives, which was isolated from an outbreak survived on concrete surfaces at 23°C for at least 21 d. It was viable for longer than the farm B clinical isolate where an outbreak was not detected. Further work is required to understand this difference. However, Grund et al. (2020) showed that EC may survive on environmental sources for up to 178 d, with inoculates kept at 15°C surviving longer than inoculates kept at 25°C and 37°C. It is possible that EC thrives better at lower temperatures while temperatures over 30°C lead to desiccation and cell death. The improved ability of Enterococcus *spp*. to survive lower temperatures has been demonstrated previously (Cools et al., 2001; del Mar Lleo, 2005; Dolka et al., 2016).

The potential of the camera technology as a tool to predict health and welfare issues in broiler flock has been investigated by other researchers (Kashiha et al., 2013; Dawkins et al., 2017; Van Hertem et al., 2018). However, the data from our study was inconclusive in detecting EC infection, primarily due to the absence of clinical symptoms of the disease in any of the trial farms; normal activity and distribution patterns of birds on farms C and D were consistent with those previously reported for healthy broiler populations (Aydin et al., 2010; Demmers et al., 2011). Nevertheless, this technology holds promise as a reduction in bird activity and change in the distribution was detected in all houses of farm B where incidental gastrointestinal health issues were diagnosed. A larger study is needed to ensure that the detected changes in bird activity and distribution is easier to detect in real time, and attributable to an EC outbreak. To be of value to producers, a future behavioral monitoring system should provide additional information over and above, and earlier than gained from monitoring feed and water consumption, and human inspection alone.

## CONCLUSIONS

Our study provides valuable new insights into EC transmission pathways and mechanisms and lays foundations for further research on EC pathogenesis and early detection of disease. This study has highlighted a range of possible environmental reservoirs for EC*,* by detecting DNA in a range of environmental sources. The results indicate that EC may be present as a harmless commensal at low levels within the farm environment, indicating further work is required to understand conditions that promote changes of this commensal to a pathogen that causes large disease outbreaks on UK farms. Future studies comparing EC isolates from farms where no outbreak was reported with isolates from outbreak farms would shed more light on properties associated with pathogenesis.

## DISCLOSURES

The authors declare no conflicts of interest.

## Appendix Supplementary materials

Image, application 1

## Footnotes

Supplementary material associated with this article can be found, in the online version, at doi:10.1016/j.psj.2024.104224.
