# Supplementary material for: Towards the identification of transmission pathways and early detection of Enterococcus cecorum infection in broiler chickens
Source: Poult Sci. 2024 Aug 16;103(11):104224. doi: 10.1016/j.psj.2024.104224 (PMC11402041; doi:10.1016/j.psj.2024.104224)
Supplement: Supplementary file 1 [file mmc1.docx]

Supplementary Table 1: List of bacterial isolates used in study. All isolates were identified using MALDI-TOF and submitted for whole genome sequencing.

| Isolate ID | Species | Source | Bird age | Isolation date | Origin | Use in study |
| --- | --- | --- | --- | --- | --- | --- |
| IS12-14619 | *E. cecorum* | Pericardial swab | 15 days | 06/07/2022 | Surveillance | Positive controls strain |
| IS12-14770 | *E. cecorum* | Joint swab | 17 days | 16/08/2022 | Surveillance | Positive controls strain |
| IS12-14580 | *E. cecorum* | Not specified | 22 days | 28/06/2022 | Surveillance | Positive controls strain |
| EC2/CEC22 | *E. cecorum* | Caecum | 21 days | 02/11/2022 | Surveillance | Survival studies |
| D22-2347-1 | *E. cecorum* | Farm B Joint fluid | 21 days | 13/12/2022 | *Post mortem* | Survival studies |
| B5B003P1_C1 | *E. faecalis* | Farm A caecum | 23 days | 20/06/2023 | Farm trial | qPCR specificity |
| ES8/23/B | *E. faecium* | Farm B caecum | NA | 17/01/2023 | Farm trial | qPCR specificity |
| ES13/23/B | *E. gallinarum* | Farm B caecum | NA | 17/01/2023 | Farm trial | qPCR specificity |
| ES16/23/B | *E. hirae* | Farm B caecum | NA | 17/01/2023 | Farm trial | qPCR specificity |
| ES12/23/B | *E. casseliflavus* | Farm B caecum | NA | 17/01/2023 | Farm trial | qPCR specificity |
| ES9/23/B | *E. durans* | Farm B caecum | NA | 17/01/2023 | Farm trial | qPCR specificity |


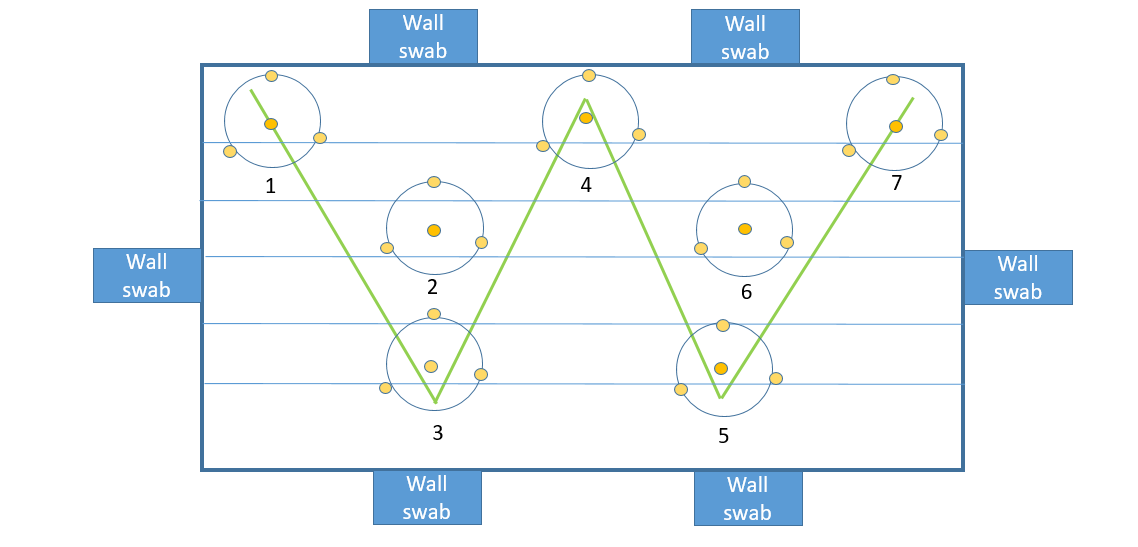


**Supplementary figure 1**. Diagram of a broiler shed showing sampling points of pooled litter samples and floor swabs (Zones 1-7) and wall swabs. Floor swabs were taken on day -1, after disinfection and before litter application. Pooled litter samples were taken on days 7, 14 and 21. Sampling of litter consisted of sampling on and around a W-shaped trajectory (green lines). Four samples (yellow dots) from each of the zones (blue circles) where collected and pooled into a single sample. Wall swabs were collected by swabbing a 50 cm2 area on day -1. The blue lines represent approximate locations of feeder and drinker lines


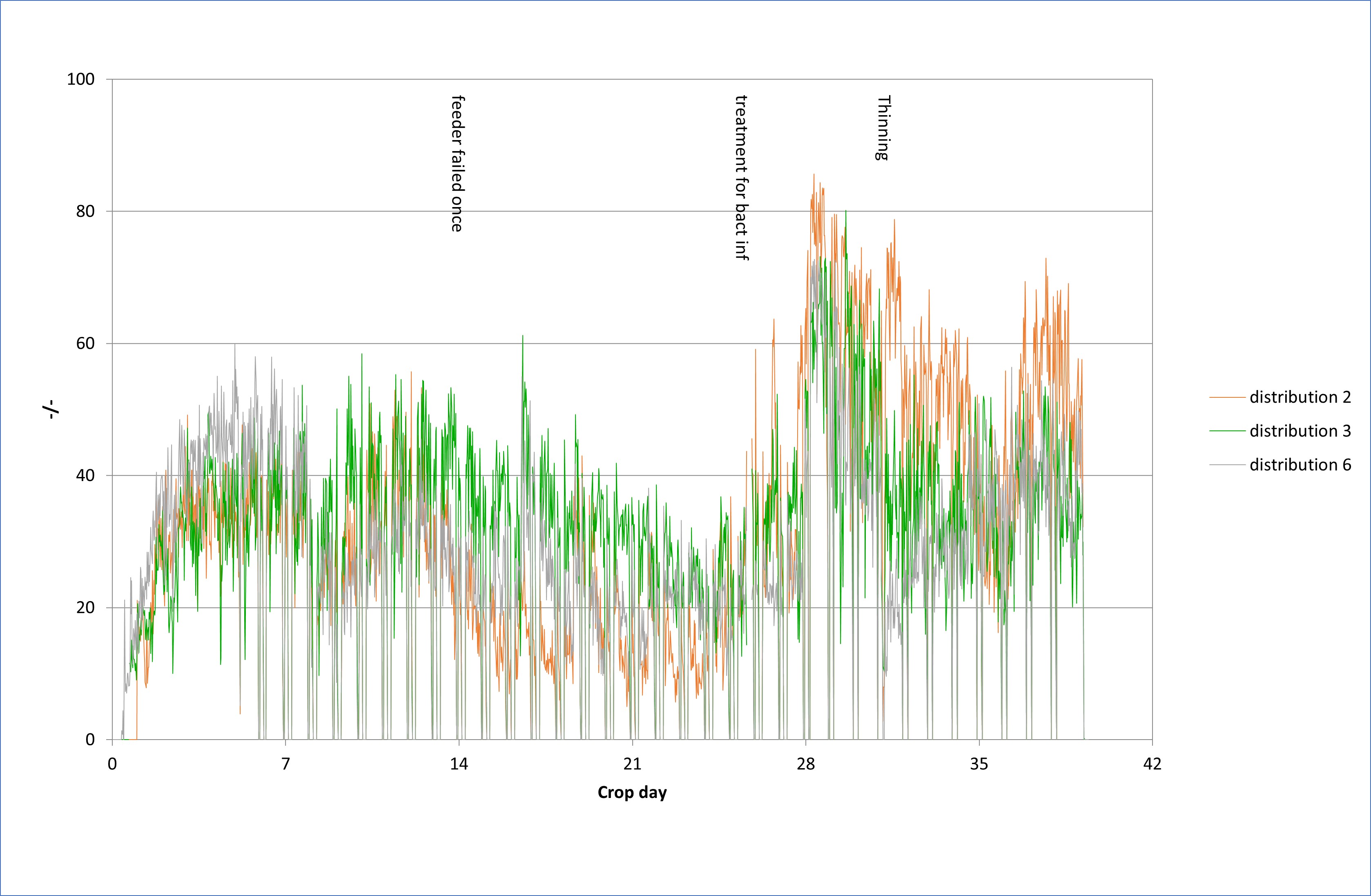


**Supplementary figure 2**. Farm B house 2 Distribution. The y-axis is a dimensionless unit: ∙/∙ . The legend refers to the three cameras in the shed that produced useable data.


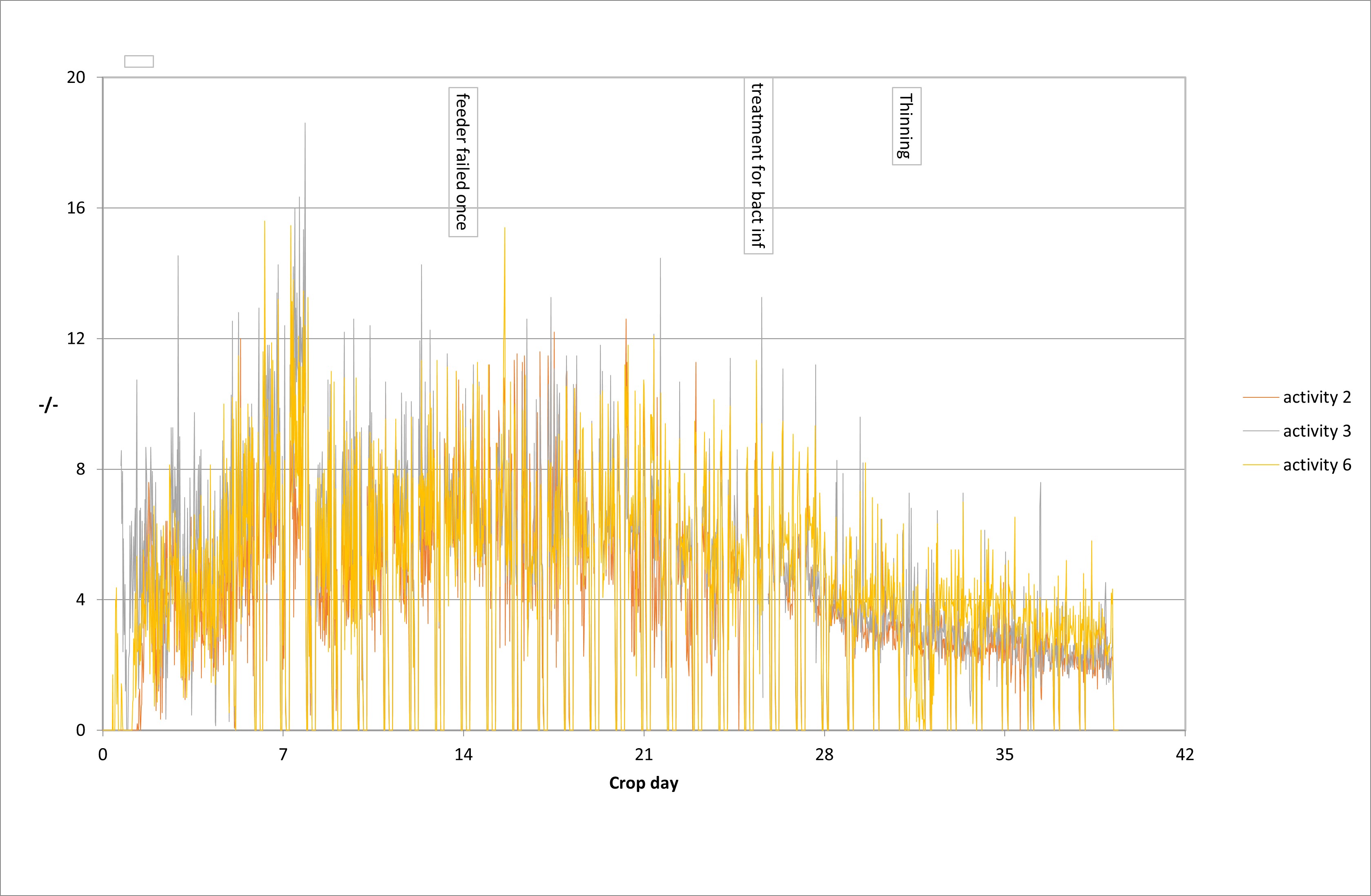


**Supplementary figure 3.** Farm B house 2 Activity. The y-axis is a dimensionless unit: ∙/∙. The legend refers to the three cameras in the shed that produced useable data.


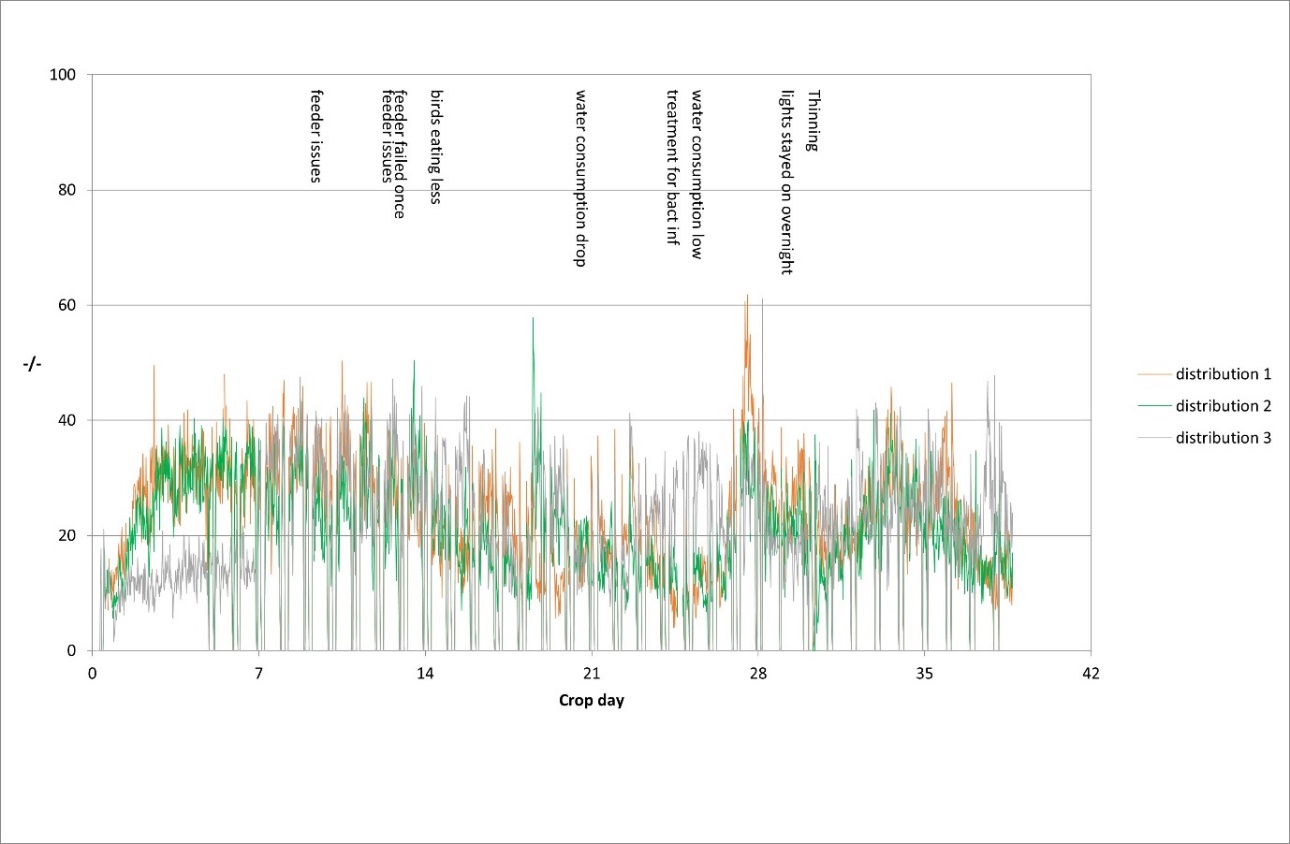


**Supplementary figure 4**. Farm B house 3 distribution. The y-axis is a dimensionless unit: ∙/∙. The legend refers to the three cameras in the shed that produced useable data.


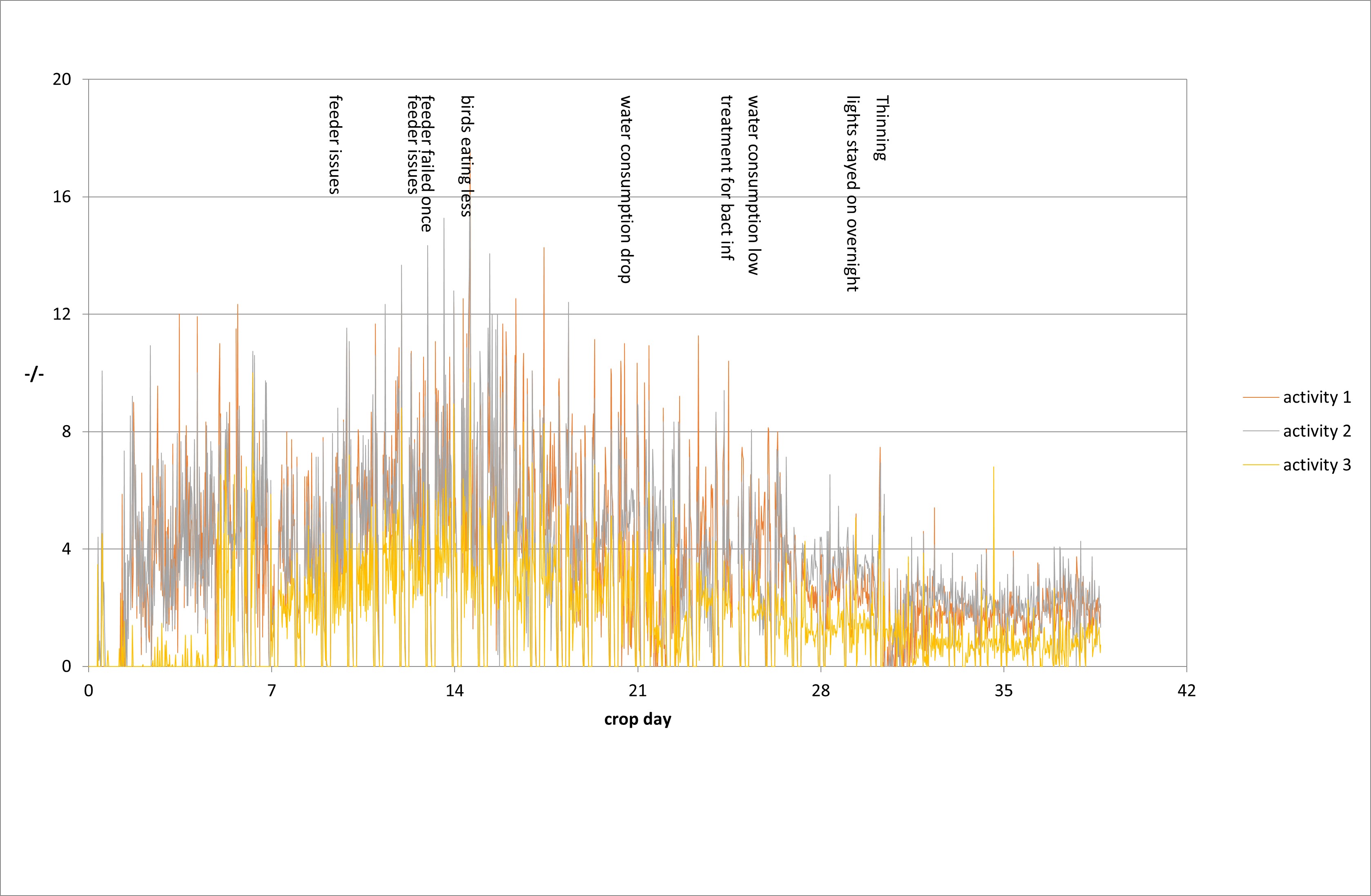


**Supplementary figure 5**. Farm B house 3 Activity. The y-axis is a dimensionless unit: ∙/∙ .The legend refers to the three cameras in the shed that produced useable data.


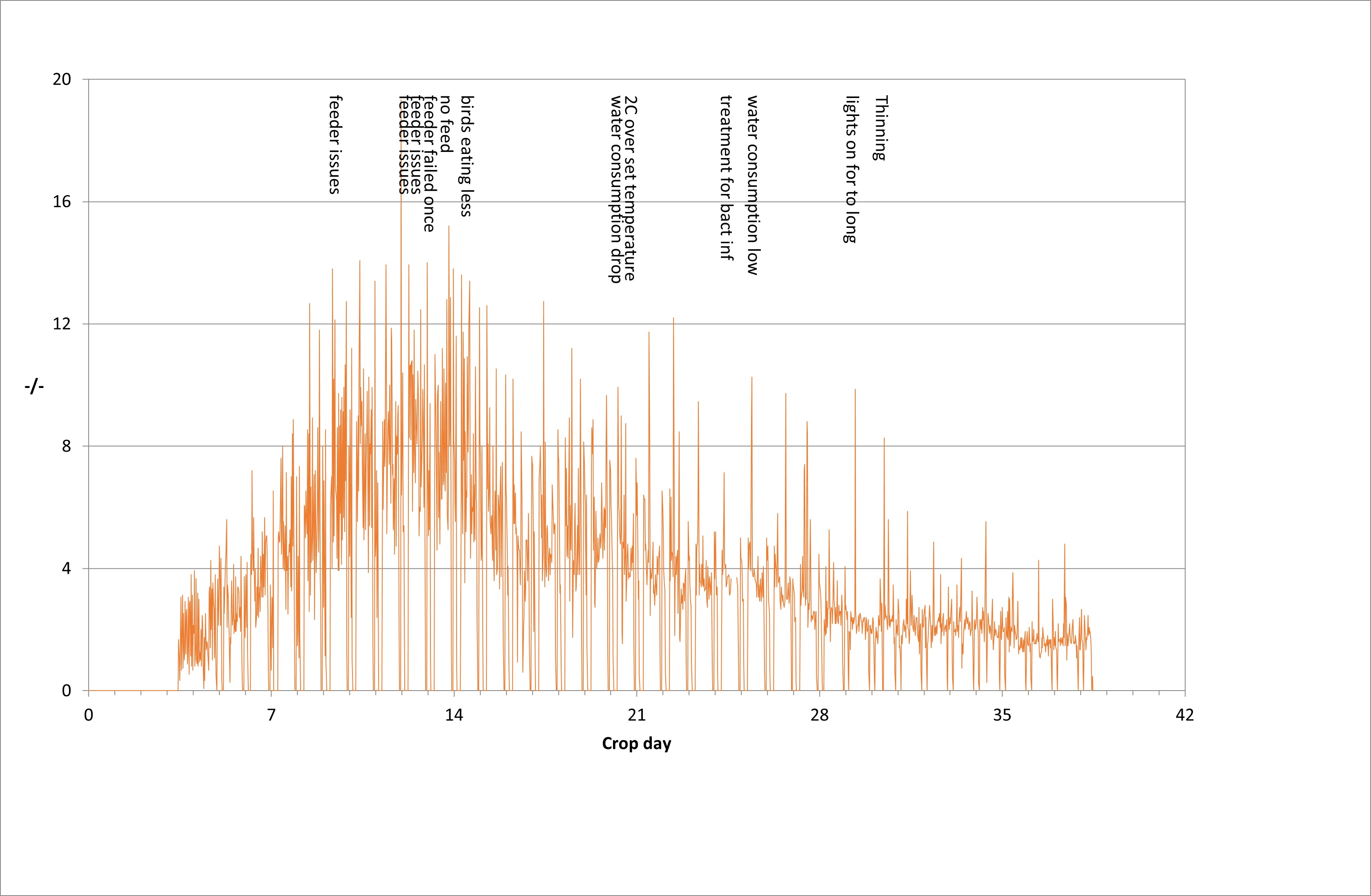


**Supplementary figure 6**. Farm B house 4 Activity. The y-axis is a dimensionless unit: ∙/∙


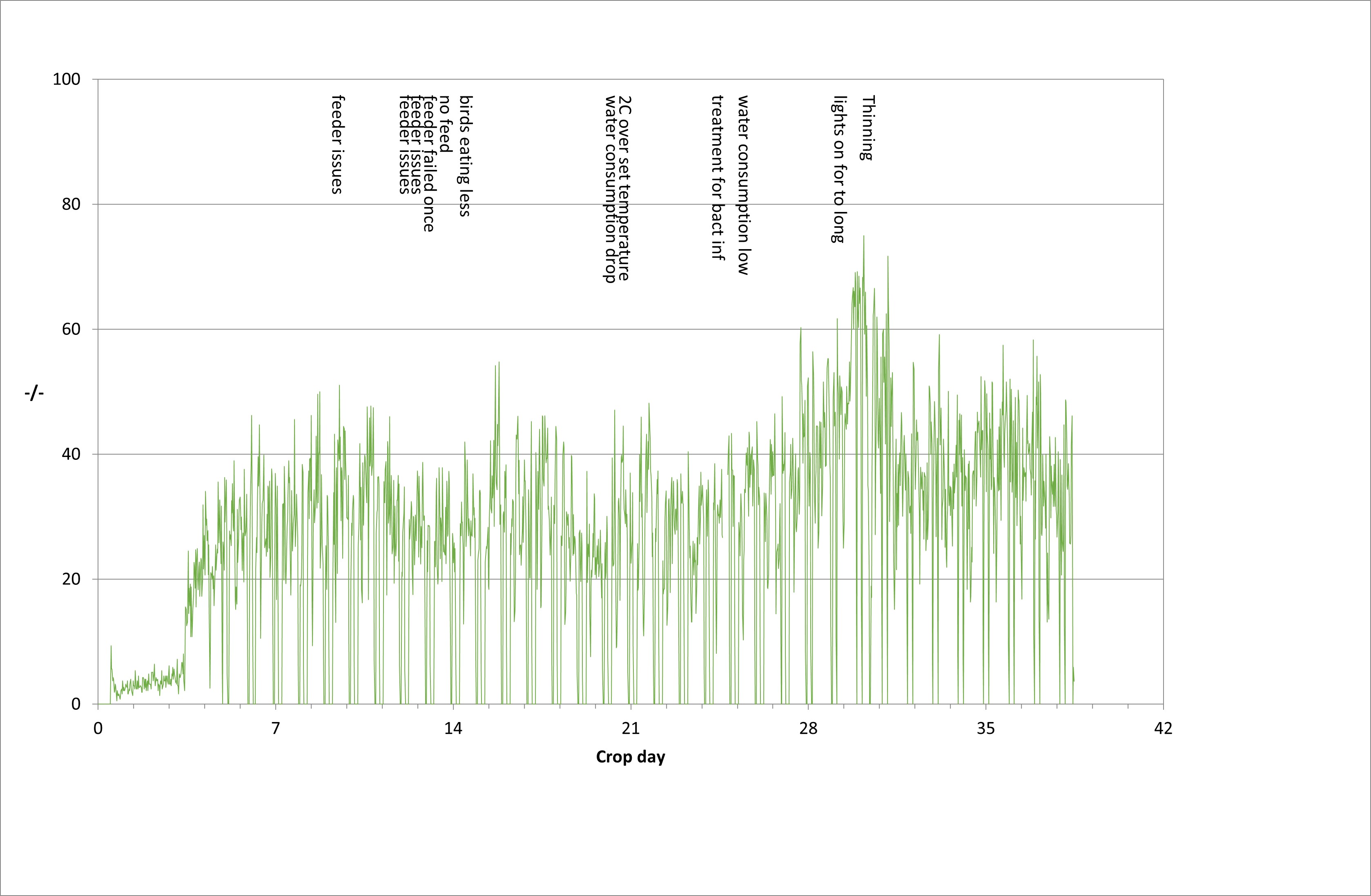


**Supplementary figure 7**. Farm B, House 4, distribution. The y-axis is a dimensionless unit: ∙/∙


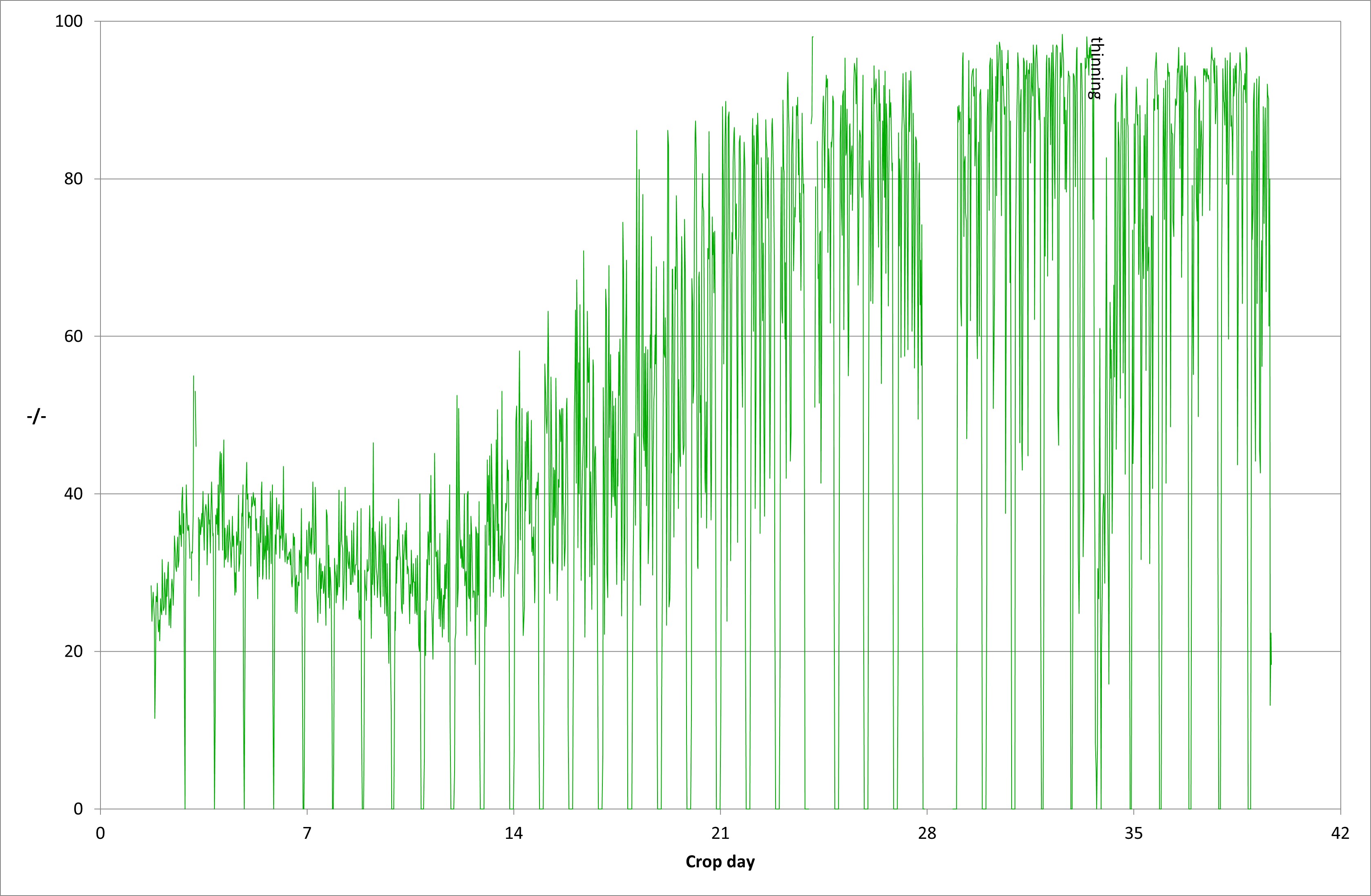


**Supplementary figure 8.** Farm D house 1 Distribution. The y-axis is a dimensionless unit: ∙/∙


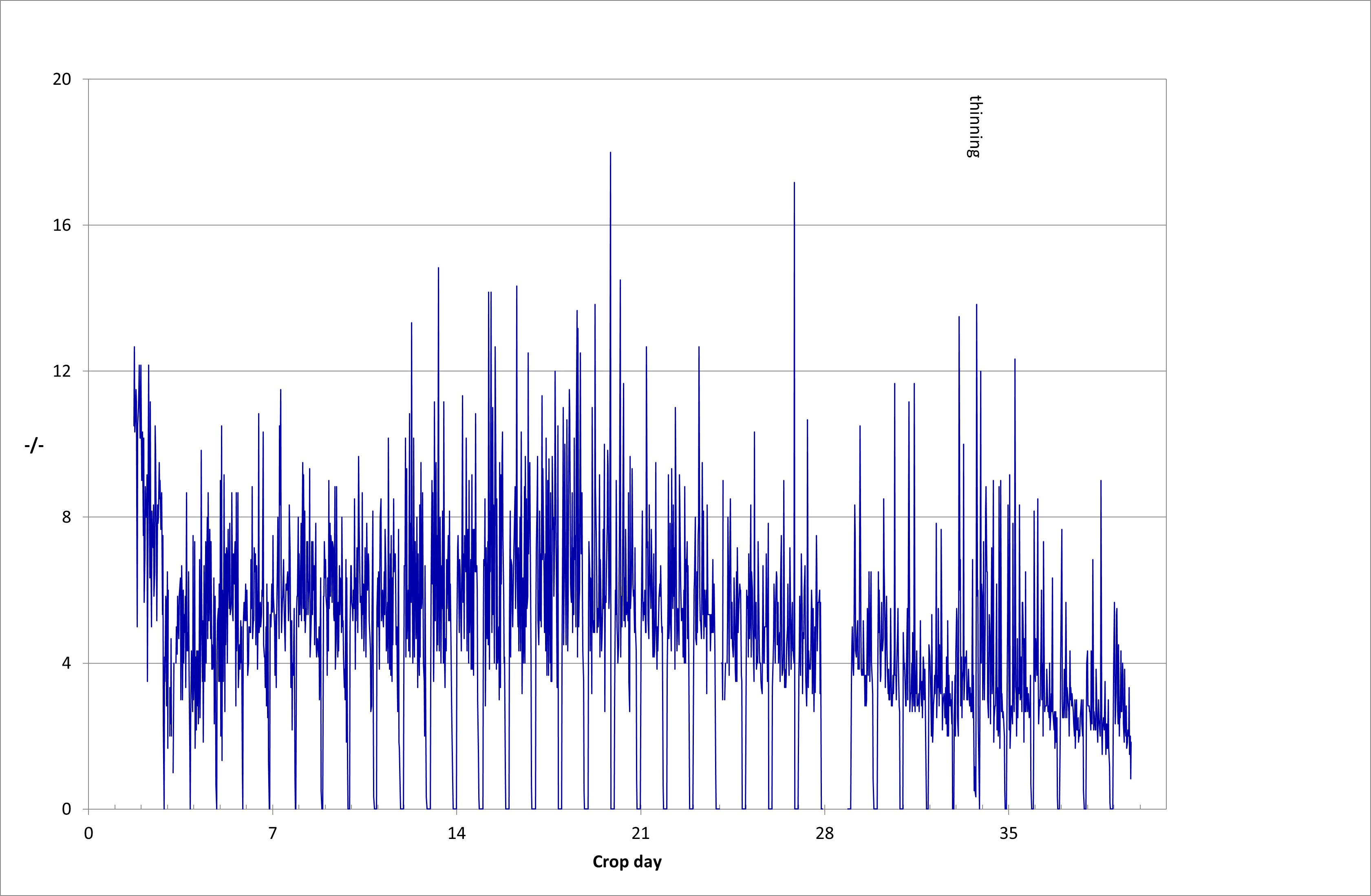


**Supplementary figure 9.** Farm D house 1 Activity. The y-axis is a dimensionless unit: ∙/∙

**Supplementary figure 10.** Farm D house 2 Activity. The y-axis is a dimensionless unit: ∙/∙


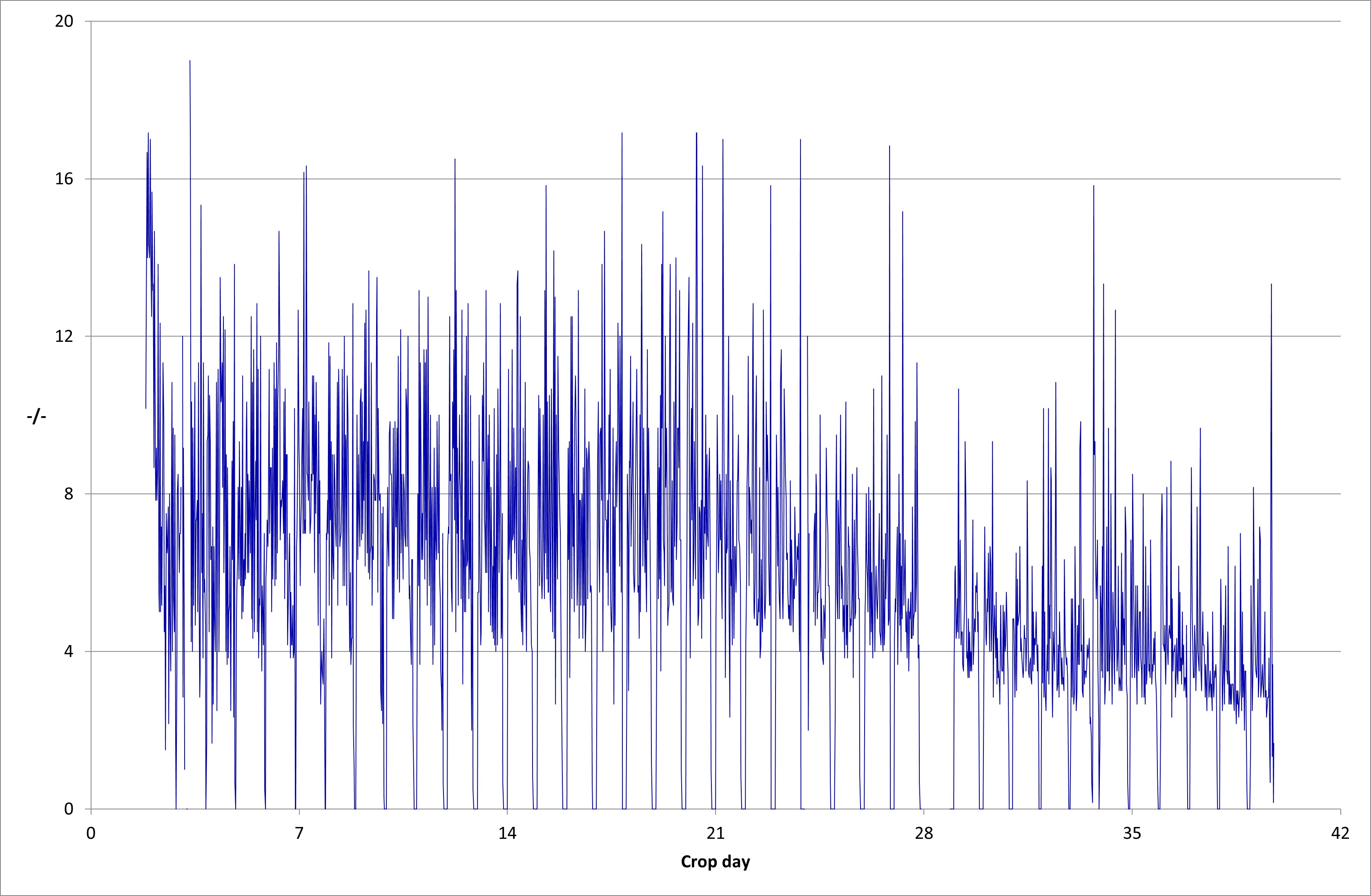

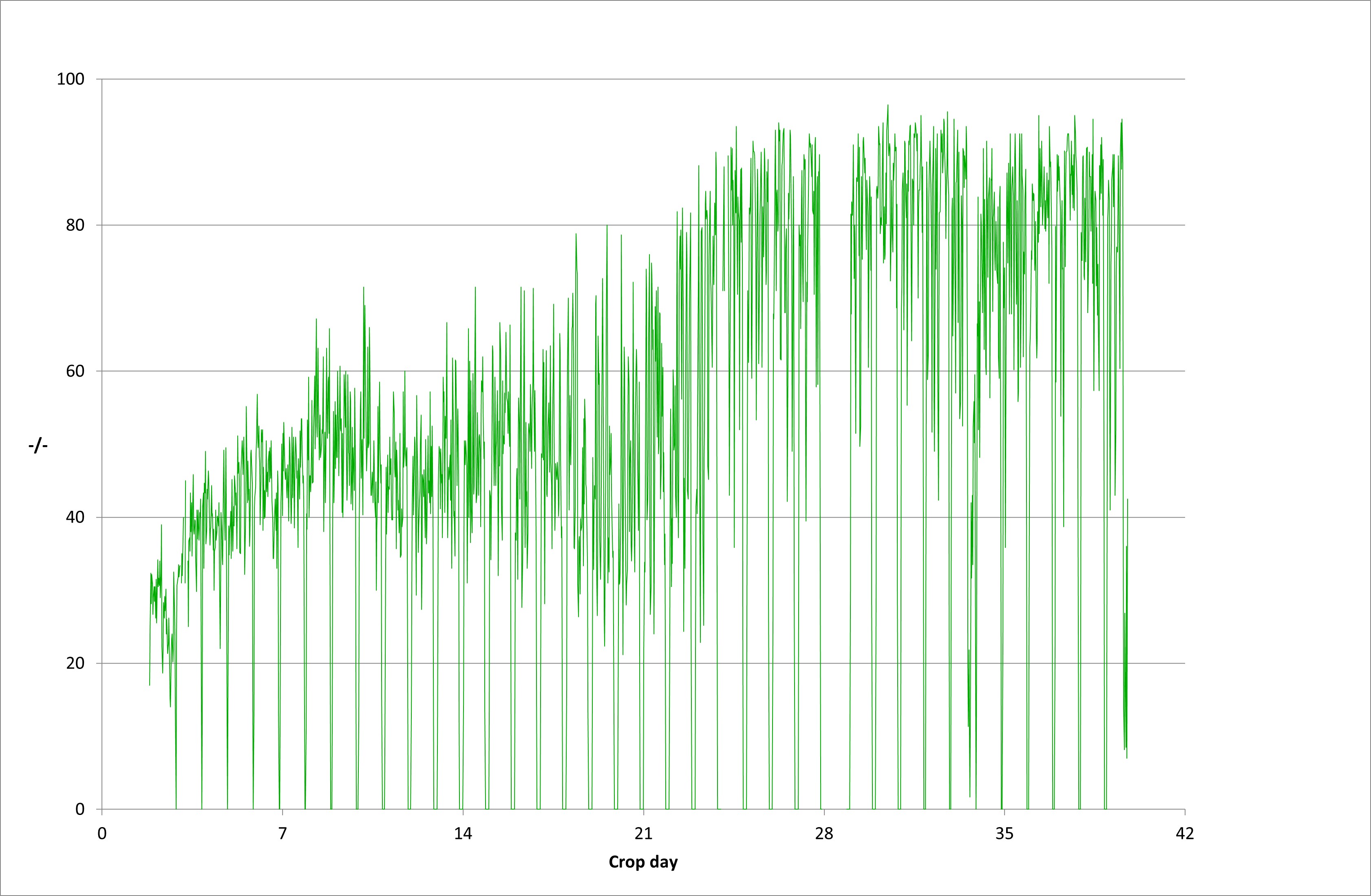


**Supplementary figure 11.** Farm D house 2 Distribution. The y-axis is a dimensionless unit: ∙/∙
